# Diffusion tensor imaging revealed different pathological processes of white matter hyperintensities

**DOI:** 10.1186/s12883-021-02140-9

**Published:** 2021-03-19

**Authors:** Zhi-gang Min, Hai-rong Shan, Long Xu, Dai-hai Yuan, Xue-xia Sheng, Wen-chao Xie, Ming Zhang, Chen Niu, Tahir Mehmood Shakir, Zhi-hong Cao

**Affiliations:** 1 Department of Radiology, The Affiliated Yixing Hospital of Jiangsu University, NO.75 Tongzhenguan Road, Yixing, Jiangsu Province 214200 P.R. China; 2 Department of Radiology, First Affiliated Hospital of Xi’an Jiaotong University, Xi’an, Shaanxi Province China

**Keywords:** White matter hyperintensities, Diffusion tensor imaging, Pathological processes

## Abstract

**Background:**

Although increasing evidence showed the correlations between white matter hyperintensities (WMHs) and cognitive impairment, the relationship between them is still modest. Many researchers began to focus on the variation caused by the heterogeneity of WMH. We tried to explore the pathological heterogeneity in WMH by using diffusion tensor imaging (DTI), so as to provide a new insight into the future research.

**Methods:**

Diffusion weighted images (DWIs) of the brain were acquired from 73 patients with WMH and 18 healthy controls, which were then modeled by DTI. We measured fractional anisotropy (FA), mean diffusivity (MD), axial diffusivity (AD), and radial diffusivity (RD) of white matter of the periventricular frontal lobe (pFL), periventricular occipital lobe (pOL), periventricular parietal lobe (pPL) and deep centrum ovales (dCO), and grouped these measures according to the Fazekas scale. Then we compared the DTI metrics of different regions with the same Fazekas scale grade.

**Results:**

Significantly lower FA values (all *p* < 0.001), and higher MD (all *p* < 0.001) and RD values (all *p* < 0.001) were associated with WMH observed in the periventricular frontal lobe (pFL) compared to all other regions with the same Fazekas grades. The AD of WMH in the pFL was higher than that of pPL and dCO, but the differences between groups was not as high as of MD and RD, as indicated by the effect size. In the normal control group, DTI metrics between pFL and other regions were not significantly different or less significant different. The difference of DTI metrics of WMH between pPL, pOL and dCO was lower than that of normal white matter, as indicated by the effect size.

**Conclusion:**

Distinct pathological processes can be revealed by DTI between frontal periventricular WMH and other regions. These processes may represent the effects of severe demyelination within the frontal periventricular WMH.

**Supplementary Information:**

The online version contains supplementary material available at 10.1186/s12883-021-02140-9.

## Background

White matter hyperintensities (WMHs), which appear hyperintense on T2-weighted imaging (T2WI) or fluid-attenuated inversion recovery (FLAIR) images, are common findings on magnetic resonance imaging (MRI) in elderly people. The prevalence of WMH increases with age and reaches 90% in the general population over 80 years old [1]. They are usually attributed to chronic small vessel ischemia. Although with similar signals on FLAIR or T2WI, their pathologic changes are heterogeneous, which include myelin pallor, enlargement of perivascular spaces, discontinuity of ependyma, infarctions, gliosis, and axonal loss [2–5]. Existing evidence shows WMH severity is associated with the risk of dementia in the general population [6]. The two most commonly-used methods for evaluating WMH severity are qualitative grading scales and quantitative WMH volumetric measurements [7, 8]. Fazekas et al. [7] categorized periventricular WMHs into three grades. Moderate and severe (grades 2 and 3) periventricular WMHs are believed to be related to ischemia, whereas mild (grade 1) periventricular WMHs are considered non-ischemic, mainly due to a partial loss of the ependymal lining [8]. However, correlations between the volume of WMHs and cognitive performance are modest, and many clinical variations cannot be explained by volumetric measures [9].

Most of the recent research showed that lesions location might be another important factor affecting cognition [10, 11]. In the frontal lobe, parieto-occipital lobe, and other parts of the brain, periventricular WMHs are involved in cognitive impairment [9, 10, 12, 13]. Carnevale et al. [10] found that alterations in specific white matter fiber-tracts are related to impaired cognition. Abnormal anterior thalamic radiation appears related to impaired memory, and the forceps minor seems to be involved in processing speed. Cremers et al. [11] suggested that the identification of tract-specific microstructural changes is helpful for understanding the mechanisms of cognitive impairment. Some studies have found that the periventricular WMHs are significantly associated with various cognitive functions, and other studies showed that subcortical WMHs are also involved; while few other studies found no associations [14, 15].

The heterogeneity of WMHs might explain why some clinical variations cannot be determined by volumetric measures of WMH [4, 16, 17]. Habes et al. [12] supported the hypothesis that different underlying pathophysiologic mechanisms influenced regional patterns of WMH distribution; they found that frontal WMHs are more strongly associated with blood pressure and cortical atrophy, while only dorsal WMHs are associated with genetic risk factors for Alzheimer s disease. However, little is known about the pathological bases of these WMHs.

Diffusion tensor imaging (DTI) is a unique tool for identifying microstructural white matter changes in vivo and may be able to characterize pathological substrates of WMH since it can detect the abnormality of white matter sensitively [18].. Studies using DTI and magnetization transfer imaging found a difference between frontal and parieto-occipital periventricular WMHs [19, 20].

We therefore hypothesized that WMHs that arose from different pathological processes would undergo different microstructural changes that could be detected by DTI. This study aimed to verify the pathological heterogeneity of WMHs by comparing DTI metrics, obtained from different brain regions, using the same grading scale. We additionally investigated microstructural changes in these brain regions.

## Methods

### Participants

The participants were local community-dwelling individuals. Our institutional review board approved this prospective study and written informed consent was acquired from each patient. Patients with WMHs confirmed by FLAIR or T2WI results were recruited. The exclusion criteria were: 1) infarction, tumor, encephalitis and other diseases affected white matter; 2) blurred images due to severe motion artifacts. Twenty-four patients with other lesions and 4 patients with severe motion artifacts were excluded. Finally, a total of 73 patients with WMHs were included in this study. There were 42 male and 31 female patients, with an average age of 72.6 years. We additionally recruited 18 (10 male, 8 female) healthy volunteers, with normal white matter with a Fazekas scale of 0. The average age of control group was 65.4 years. The Fazekas scale was adopted to classify periventricular and deep WMHs as grades 1, 2, or 3. In periventricular white matter, “caps” or pencil-thin lining WMH were classified as grade 1, smooth “halo” WMH were classified as grade 2 and irregular WMH extending into the deep white matter were classified as grade 3; in deep white matter punctate foci WMH were classified as grade 1, beginning confluence of foci WMH were classified as grade 2 and large confluent areas were classified as grade 3 [7]. All grades were calculated separately from areas within the frontal and parietal-occipital lobes. Two neuro-radiologists assessed each patient, and conflicting results were discussed until an agreement was made.

### DTI and post-processing

**Fig. 1:**
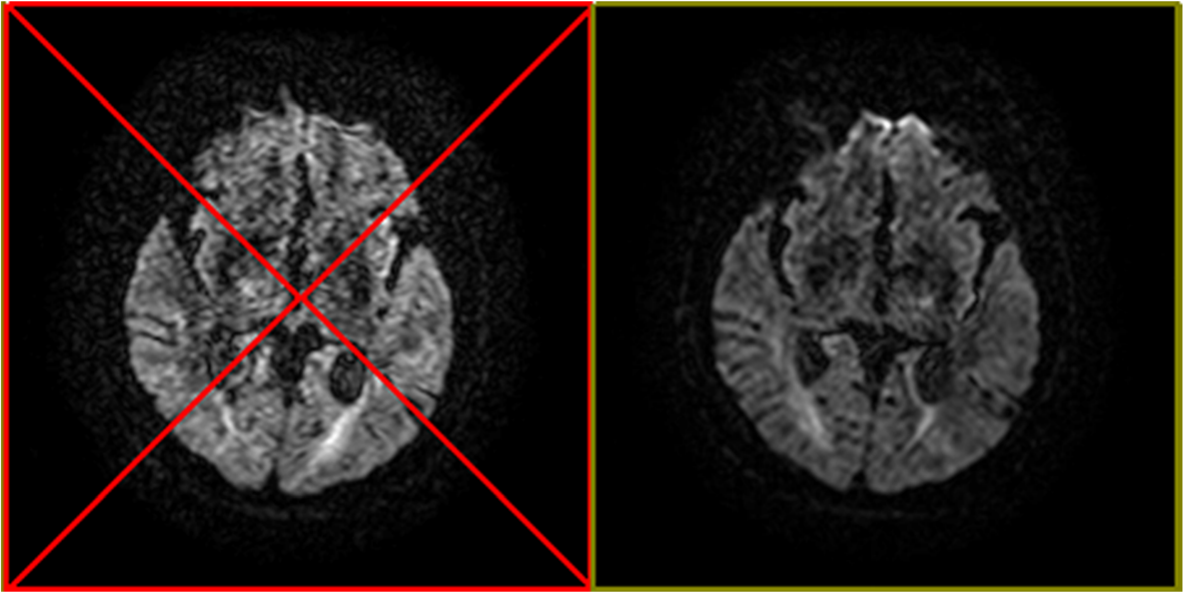
Corrupted images will be eliminated with automatic outlier slice rejection if its error-area is bigger than 3%. Left image with red “X” were excluded as corrupted image

Diffusion weighted images (DWIs) were acquired from all patients and controls using a 3.0-T MR scanner (Philips Achieva, Best, the Netherlands) equipped with a 16-channel head-neck phased array coil. The imaging parameters were as follows: slice thickness of 2 mm with no gaps, repetition time of 8000 ms, echo time of 87 ms, field-of-view 228 mm × 228 mm, 32 DWIs (b-values = 1000s/mm^2^) with diffusion gradients along different directions and 1 non-DWIs (b-value = 0) were acquired, an acquisition matrix of 112 × 112 was interpolated to an image matrix of 224 × 224. A total of 60 consecutive slices were acquired over 6 min. The raw data are exported to DTIStudio (Johns Hopkins University; https://www.mristudio.org/) [21]. Using the Automated Image Registration (AIR) tool, we used an affine warp model to correct image distortions due to eddy currents and misregistration errors due to head motion. After AIR-based image co-registration, rotational operation is extracted and the orientation of the b-vectors was corrected. Then tensor was estimated with linear least square fitting, automatic outlier slice rejection was performed with relative error > 3% to eliminate the corrupted images of error area more than 3%. Exclusion of corrupted images (with red “X” in the inspection window) can be verified with “Original View” (Fig. 1). Maps of fractional anisotropy (FA), mean diffusivity (MD), axial diffusivity (AD) and radial diffusivity (RD) maps were calculated.

### Measurements

**Fig. 2:**
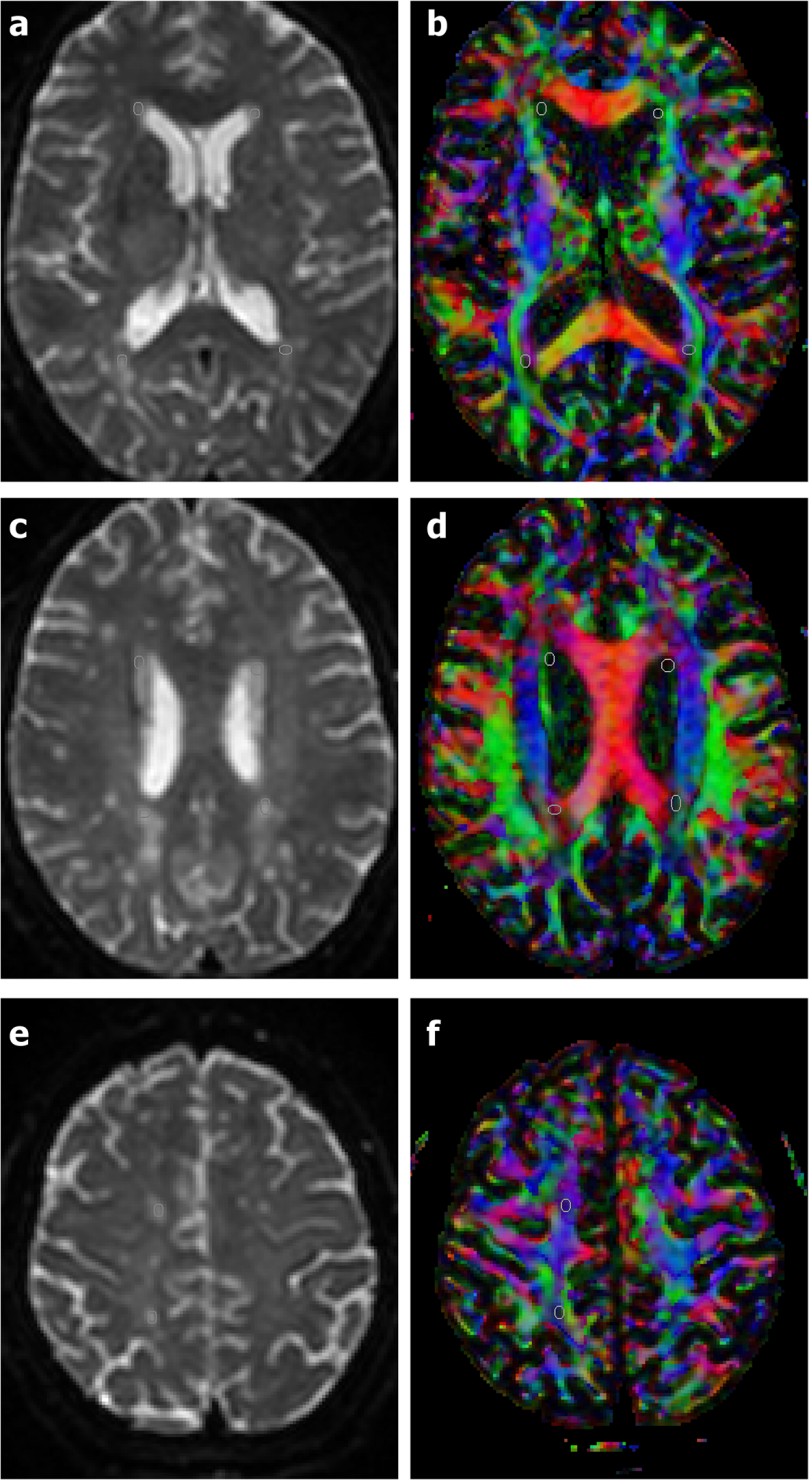
The regions of interest (ROIs) are determined on the B0 image (1**a**, 1**c**, 1**e**), and the corresponding colored FA maps (1**b**, 1**d**, 1**f**) are used to observe whether the ROIs are located in the same white matter bundle. ROIs of the periventricular frontal lobe (pFL) were located in the anterior thalamic radiation and superior fronto-occipital fasciculus. ROIs of the periventricular occipital lobe (pOL) were located in the tapetum and the posterior region of corona radiata. ROIs of the periventricular parietal lobe (pPL) were located in the junction of corpus callosum and parietal superior region of internal capsule. ROIs of deep white matter of centrum ovale (dCO) were located in the centrum ovale of frontal lobe and parietal lobe respectively

The FA, MD, AD, and RD values of WMH were measured with regions of interest (ROIs) in three periventricular regions including frontal, occipital and parietal lobes, as well as the deep centrum ovales. These areas were the most common locations of WHMs. According to the range of lesions, 2–4 ROIs were determined, and the average value was calculated. The same DTI metrics in the healthy control group were also measured in the identical white matter tract, which was controlled by using colored FA maps (Fig. 2). Mean diffusion-weighted imaging maps were used to keep the cerebral spinal fluid outside the ROI.

### Statistical analysis

All statistical analyses were performed using SPSS software (version 22, IBM, Armonk, NY, USA), and two-sided *p* values < 0.05 indicated a significant difference. Spearman correlation analysis was used to explore the relationship between DTI parameters and Fazekas scale scores. We used univariate analysis of variance (ANOVA) process of the general linear model to identify differences of FA, MD, AD, and RD values in WMH with the same Fazekas grade among regions of periventricular frontal lobe (pFL), periventricular occipital lobe (pOL), periventricular parietal lobe (pPL) and deep centrum ovales (dCO). Sex and age were used as covariates. Multiple comparisons were performed and corrected with Bonferroni. We calculated η^2^ as effect sizes (ES) of ANOVA and Hedges’g as effect sizes of multiple comparison. Box plots were generated by using GraphPad Prism 8.0 (GraphPad Software Inc., San Diego, CA, USA).

## Results

**Fig. 3:**
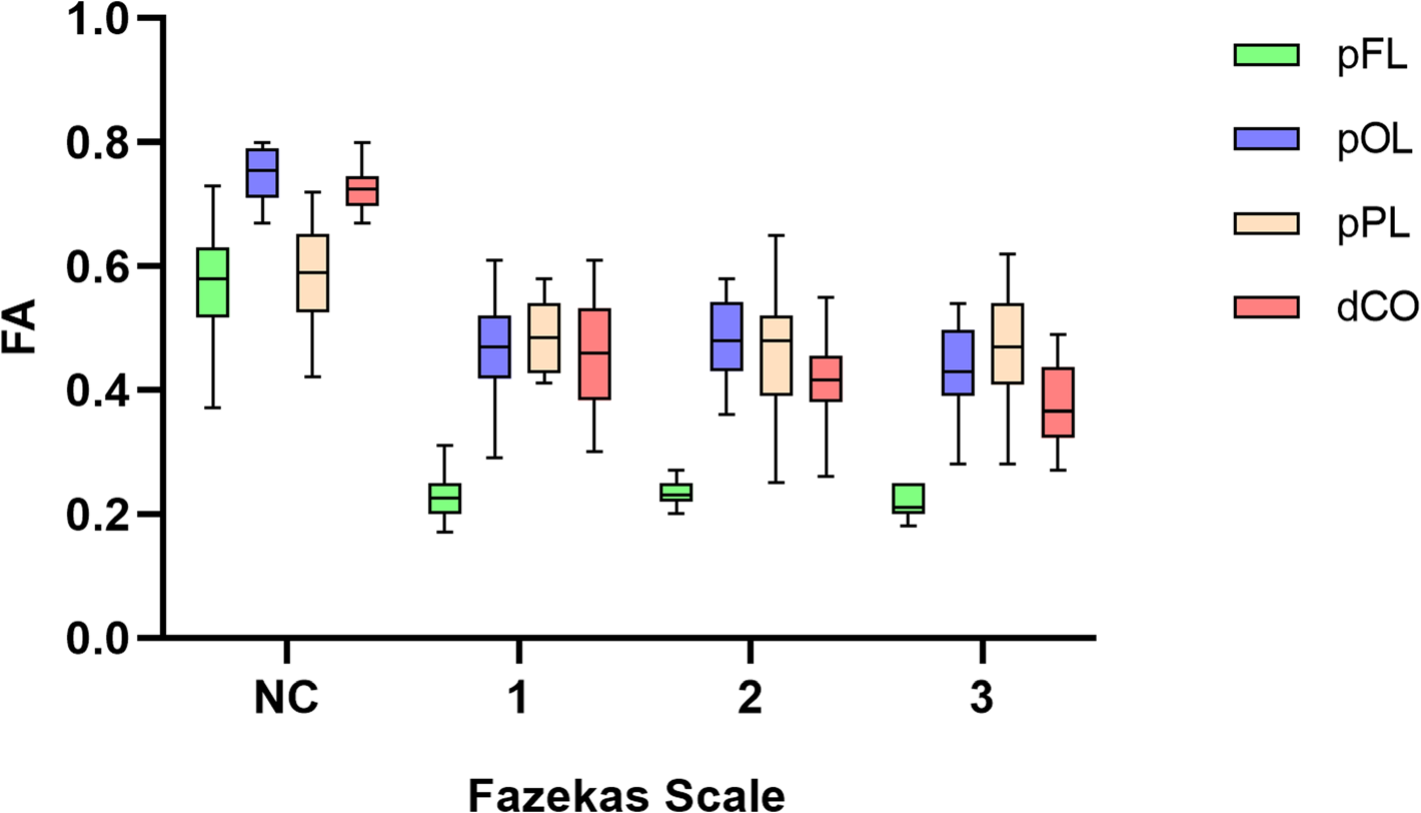
Box plot of FA with normal control and Fazekas scale 1–3 in different regions

**Fig. 4:**
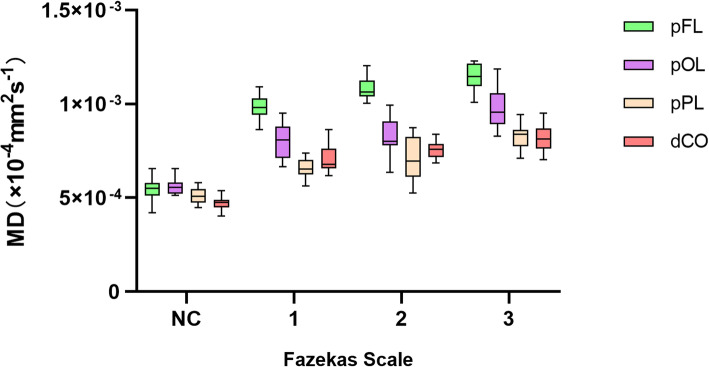
Box plot of MD with normal control and Fazekas scale 1–3 in different regions

**Fig. 5:**
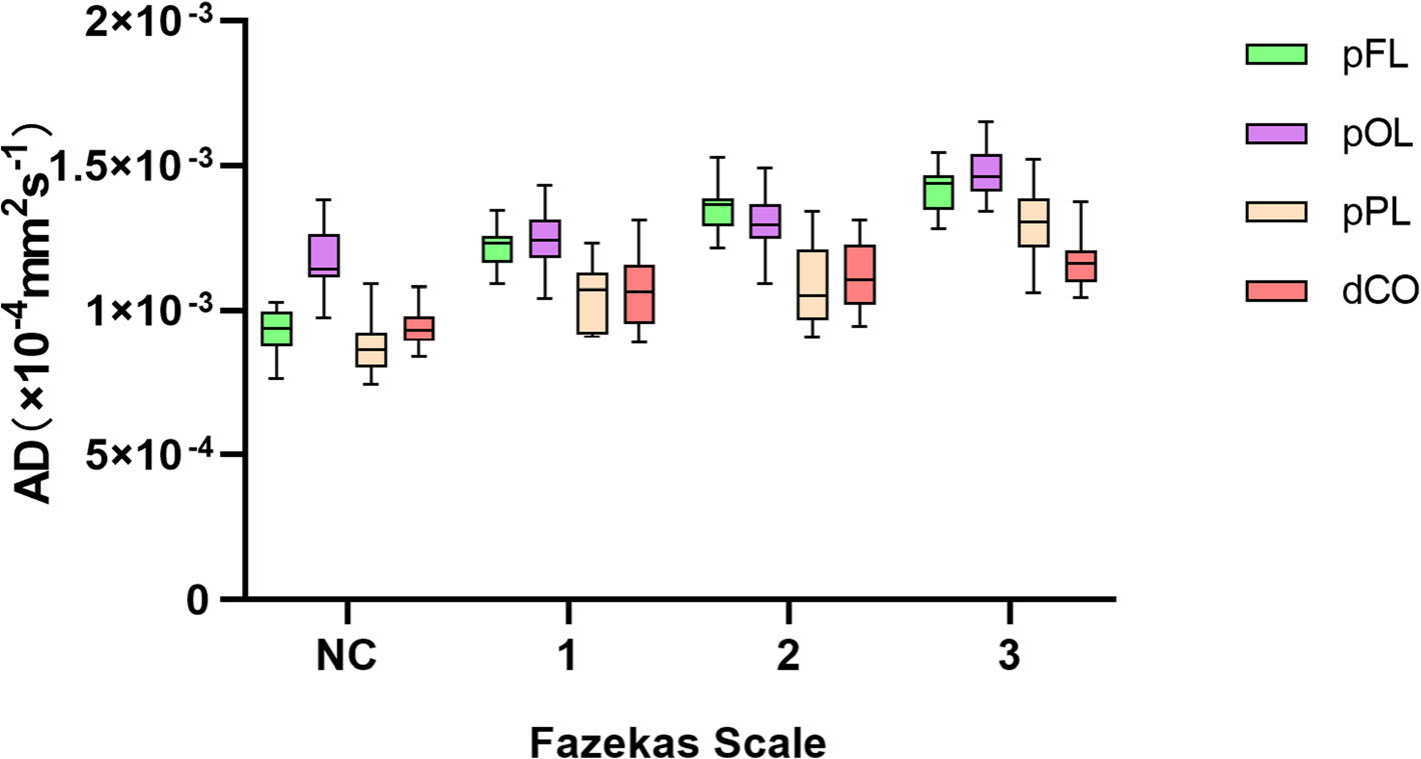
Box plot of DA with normal control and Fazekas scale 1–3 in different regions

**Fig. 6:**
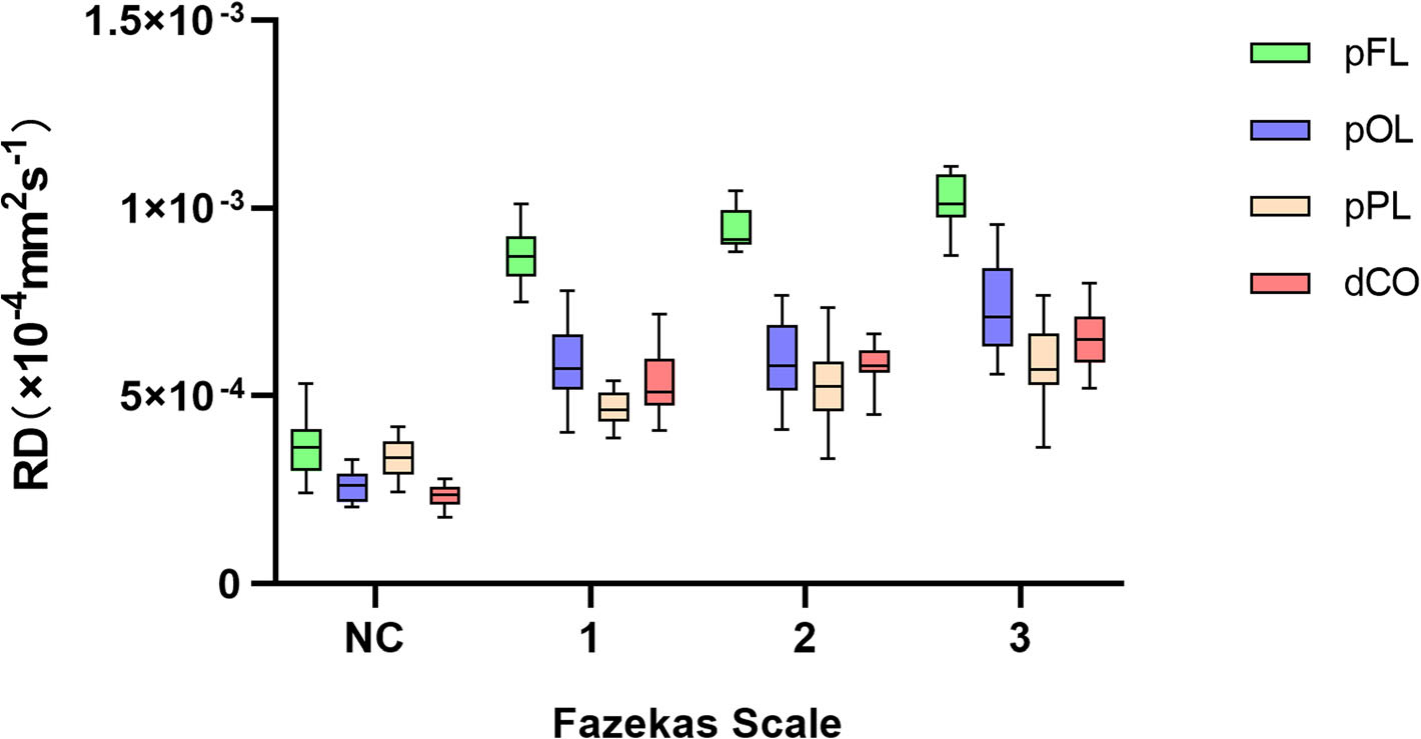
Box plot of RD with normal control and Fazekas scale 1–3 in different regions

There were 86 WMHs of Fazekas grades 1 in 46 patient, 64 WMHs of Fazekas grades 1 in 35 patients and 86 WMHs of Fazekas grades 3 in 28 patients. With increasing Fazekas grades, the DTI metrics of WMHs in different regions showed similar changes. FA values decreased, while MD, AD and RD values increased as the Fazekas grade increased (Figs.3, 4, 5, and 6). Spearman correlation analysis showed that FA, MD, AD, and RD values in all regions were significantly correlated (*p* < 0.001) with Fazekas grades (Table 1). However, there are some differences in the correlation coefficients among pFL, pOL, pPL and dCO.

**Table 1:** Correlation coefficients between DTI metrics and Fazekas scale in different regions

	pFL	pOL	pPL	dCO
FA	−0.525	−0.648	− 0.417	−0.728
MD	0.864	0.823	0.861	0.836
AD	0.891	0.749	0.831	0.666
RD	0.822	0.768	0.756	0.811

*p-*value all < 0.001. *pFL* periventricular frontal lobe, *pOL* periventricular occipital lobe, *pPL* periventricular parietal lobe, *dCO* deep centrum ovale

**Fig. 7:**
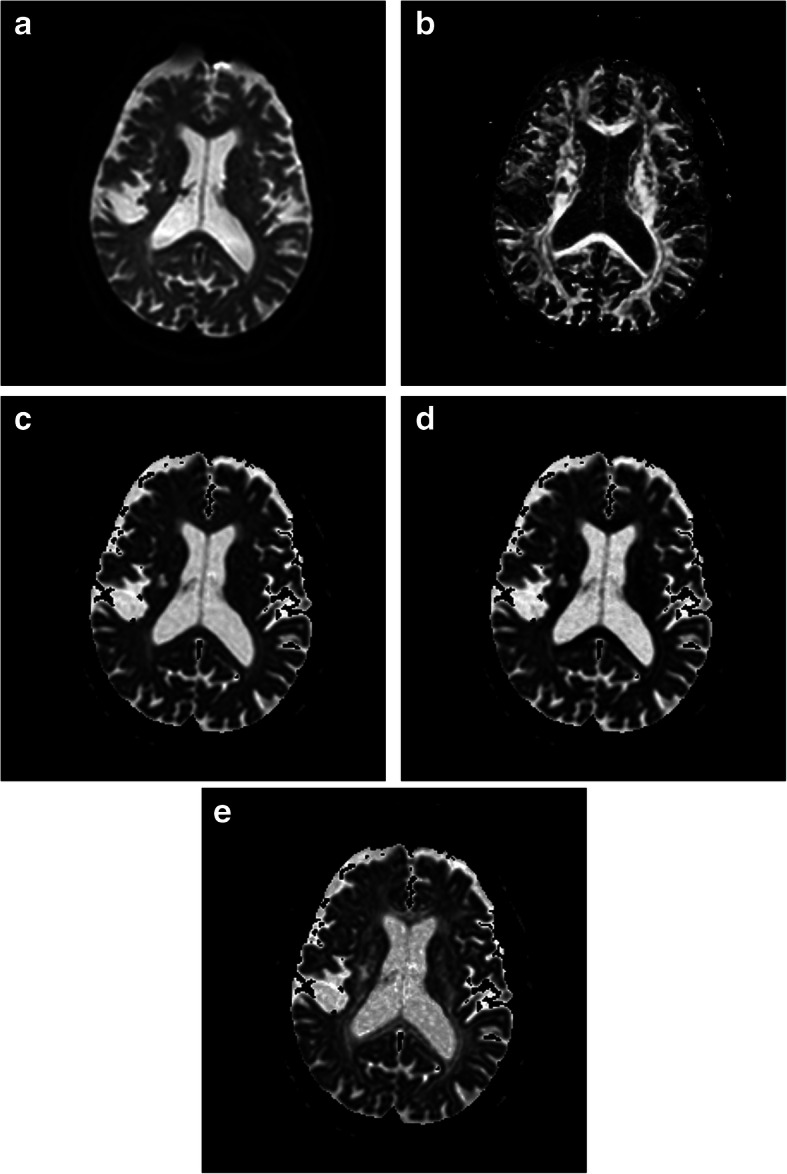
The b0 of the DWI (**a**) of a 75 years old male showed WMH (Fazekas scale 1) in the pFL, pOL, pPL and dCO. FA (**b**) of WMH in the pFL are lower than that of pOL, pPL and dCO. MD (**c**) and RD (**d**) of WMH in the pFL are higher than that of pOL, pPL and dCO. AD (**e**) of WMH in the pFL are close to that of pOL, pPL and dCO

**Fig. 8:**
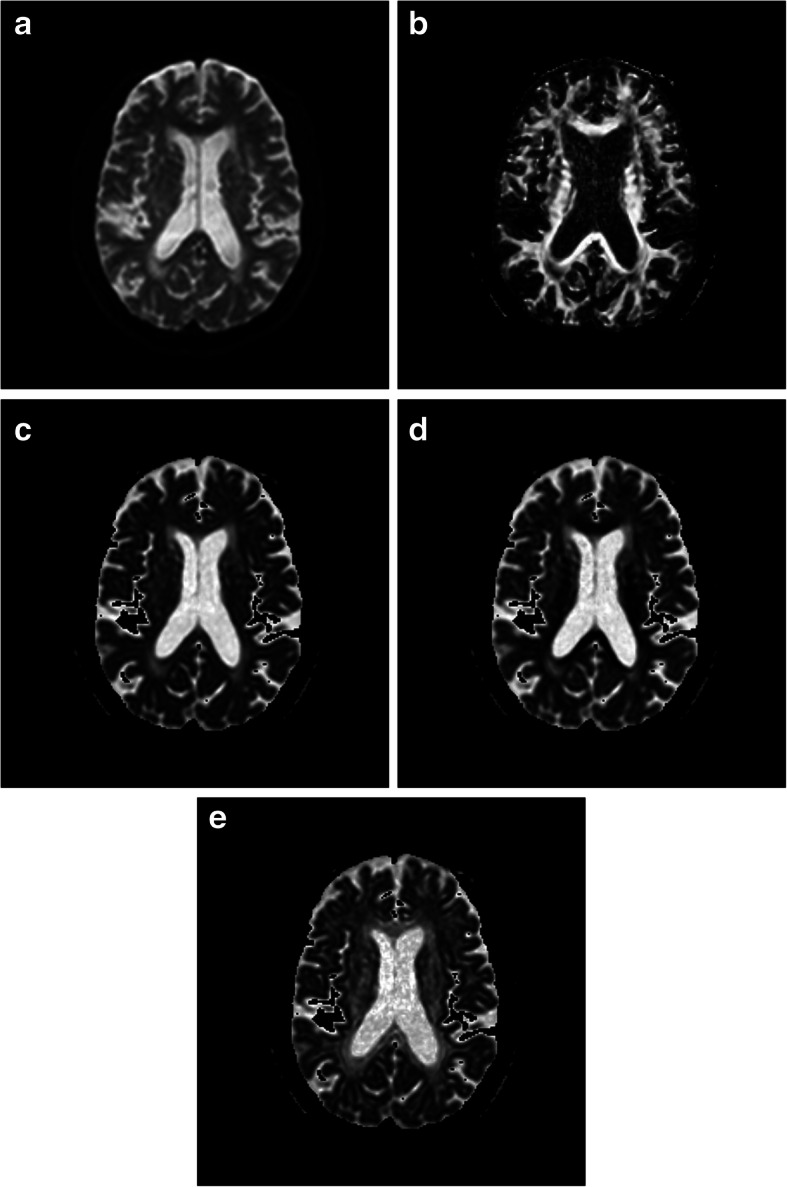
The b0 of the DWI (**a**) of a 77 years old male showed WMH (Fazekas scale 2) in the pFL, pOH, pPL and dCO. FA (**b**) of WMH in the pFL are lower than that of pOL, pPL and dCO. MD (**c**) and RD (**d**) of WMH in the pFL are higher than that of pOL, pPL and dPCO. AD (**e**) of WMH in the pFL are close to that of pOL, pPL and dCO

**Fig. 9:**
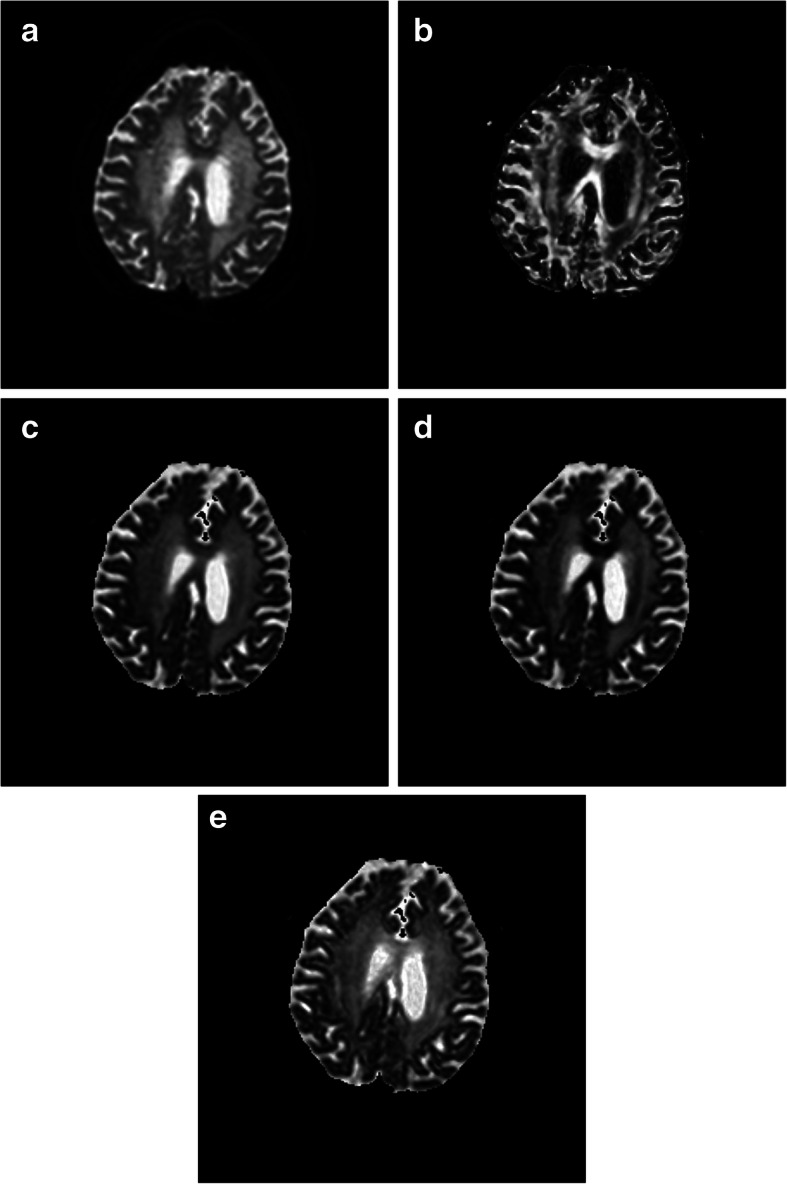
The b0 of the DWI (**a**) of a 83 years old female showed WMH (Fazekas scale 3) in the pFL, pOL and dCO. FA (**b**) of WMH in the pFL are lower than that of pPL and dCO. MD (**c**) and RD (**d**) of WMH in the pFL are higher than that of pPL and dCO. AD (**e**) of WMH in the pFL are close to that of pPL and dCO

Although DTI metrics in different regions showed similar changes, they don’t change at the same rate. FA, MD, AD and RD values of pFL showed more rapid changes from Fazekas scale 0 to 1 than other regions (Figs. 3, 4, 5 and 6). In normal control group, FA values of pFL were significantly lower than that of pOL (*p <* 0.001, ES = 2.47) and dCO (*p <* 0.001, ES = 2.22), but had no significant difference with pPL. However, FA values of the WMH with Fazekas scale 1 of pFL were significantly lower than pOL (*p <* 0.001, ES = 4.20), pPL (*p <* 0.001, ES = 6.55) and dCO (*p <* 0.001, ES = 3.65) all (Table 2). Similarly, MD and RD of the WMH with Fazekas scale 1 of pFL were significantly higher than that of all other three regions (Fig. 7). Though MD of normal white matter of pFL was only higher than dCO, and RD of normal white matter of pFL was only higher than pOL and dCO (Tables 3 and 5). In pFL, the increase of AD was lower than that of RD and MD, and it didn’t exceed pOL in Fazekas 1 (Table 3). Results of DTI metrics of WMH with grade 2 and 3 between pFL and other regions were same as those of grade 1 (Tables 2, 3, 4, and 5) (Figs. 8 and 9).

**Table 2:** Comparison of FA values of normal white matter or WMH with same Fazekas scale in different regions

	pFL Mean ± SD	pOL Mean ± SD	pPL Mean ± SD	dCO Mean ± SD	*p*-value	Effect Size (η^2^)
Normal white matter	0.56 ± 0.10	0.75 ± 0.04^**^	0.59 ± 0.08	0.72 ± 0.04^**^	< 0.001	0.566
WMH of Fazekas scale 1	0.22 ± 0.04	0.46 ± 0.09^**^	0.49 ± 0.06^**^	0.45 ± 0.09^**^	< 0.001	0.762
WMH of Fazekas scale 2	0.23 ± 0.02	0.48 ± 0.07^**^	0.46 ± 0.10^**^	0.41 ± 0.08^**^	< 0.001	0.720
WMH of Fazekas scale 3	0.21 ± 0.03	0.43 ± 0.07^**^	0.47 ± 0.10^**^	0.39 ± 0.11^**^	< 0.001	0.681

*pFL* periventricular frontal lobe, *pOL* periventricular occipital lobe, *pPL* periventricular parietal lobe, *dCO* deep centrum ovale. There was significant difference compared with pFL, *p < 0.05, **p < 0.001

**Table 3:** Comparison of MD values (×10^−4^mm^2^s^−1^) of normal white matter or WMH with same Fazekas scale in different regions

	pFL Mean ± SD	pOL Mean ± SD	pPL Mean ± SD	dCO Mean ± SD	*p*-value	Effect Size (η2)
Normal white matter	5.48 ± 0.58	5.62 ± 0.48	5.11 ± 0.40	4.69 ± 0.31^**^	< 0.001	0.436
WMH of Fazekas scale 1	9.87 ± 0.57	8.03 ± 0.84^**^	6.59 ± 0.55^**^	7.08 ± 0.71^**^	< 0.001	0.762
WMH of Fazekas scale 2	10.81 ± 0.56	8.25 ± 0.96^**^	7.13 ± 1.05^**^	7.55 ± 0.47^**^	< 0.001	0.797
WMH of Fazekas scale 3	11.48 ± 0.71	9.81 ± 1.00^**^	8.28 ± 0.68^**^	8.20 ± 0.66^**^	< 0.001	0.691

*pFL* periventricular frontal lobe, *pOL* periventricular occipital lobe, *pPL* periventricular parietal lobe, *dCO* deep centrum ovale. There was significant difference compared with pFL, *p < 0.05, **p < 0.001

**Table 4:** Comparison of AD values (×10^−4^mm^2^s^− 1^) of normal white matter or WMH with same Fazekas scale in different regions

	pFL Mean ± SD	pOL Mean ± SD	pPL Mean ± SD	dCO Mean ± SD	*p*-value	Effect Size (η2)
Normal white matter	9.11 ± 0.82	11.70 ± 1.15^**^	8.63 ± 0.84	9.41 ± 0.64	< 0.001	0.518
WMH of Fazekas scale 1	12.16 ± 0.62	12.37 ± 1.12	10.44 ± 1.19^**^	10.62 ± 1.23^**^	< 0.001	0.438
WMH of Fazekas scale 2	13.48 ± 0.71	12.96 ± 1.03	10.92 ± 1.41^**^	11.18 ± 1.14^**^	< 0.001	0.506
WMH of Fazekas scale 3	14.10 ± 0.78	14.76 ± 0.91	12.96 ± 1.32^*^	11.57 ± 0.75^**^	< 0.001	0.556

*pFL* periventricular frontal lobe, *pOL* periventricular occipital lobe, *pPL* periventricular parietal lobe, *dCO* deep centrum ovale. There was significant difference compared with pFL, *p < 0.05, **p < 0.001

**Table 5:** Comparison of RD values (×10^−4^mm^2^s^−1^) of normal white matter or WMH with same Fazekas scale in different regions

	pFL Mean ± SD	pOL Mean ± SD	pPL Mean ± SD	dCO Mean ± SD	p-value	Effect Size (η2)
Normal white matter	3.63 ± 0.79	2.59 ± 0.41^**^	3.35 ± 0.53	2.33 ± 0.30^**^	< 0.001	0.531
WMH of Fazekas scale 1	8.72 ± 0.64	5.86 ± 1.05^**^	4.66 ± 0.50^**^	5.31 ± 0.80^**^	< 0.001	0.795
WMH of Fazekas scale 2	9.47 ± 0.54	5.89 ± 1.07^**^	5.23 ± 1.16^**^	5.79 ± 0.57^**^	< 0.001	0.820
WMH of Fazekas scale 3	10.18 ± 0.72	7.33 ± 1.18^**^	5.88 ± 1.00^**^	6.52 ± 0.79^**^	< 0.001	0.723

*pFL* periventricular frontal lobe, *pOL* periventricular occipital lobe, *pPL* periventricular parietal lobe, *dCO* deep centrum ovale. There was significant difference compared with pFL, *p < 0.05, **p < 0.001

The difference of DTI metrics of WMH between pPL, pOL and dCO was lower than that of normal white matter. In normal white matter, FA values of pPL were significantly lower than that of pOL (*p* < 0.001, ES = 2.36) and dCO (*p* < 0.001, ES = 2.08). There was no significant difference in FA of WMH between pPL, pOL and dCO except for the WMH with Fazekas scale 3 between pPL and dCO (*p* < 0.001, ES = 1.20). MD of normal white matter in pPL were significantly lower than that in pOL (*p* < 0.05, ES = 1.14) and higher than dCO (*p* < 0.05, ES = 1.16). MD of WMH in pPL were still significantly lower than that in pOL (*p* < 0.001, ES = 1.90, 1.12 and 1.78), but were not significantly different with dCO. AD of normal white matter and WMH in pPL were all significantly lower than that in pOL (*p* < 0.001, ES = 3.05, 1.69, 1.67 and 1.58), but were not significantly different with dCO except for the WMH with Fazekas scale 3 (*p* < 0.001, ES = 1.30). In normal controls, RD of pPL were significantly higher than that of pOL (*p* < 0.001, ES = 1.61) and dCO (*p* < 0.001, ES = 2.38). RD of WMH with Fazekas scale 1 and 3 in pPL were significantly lower than that in pOL(*p* < 0.05, ES = 1.31; *p* < 0.001, ES = 1.33). No significant difference was found in FA of WMH between pPL and dCO.

## Discussion

Correlation analysis revealed the relationship between DTI metrics and Fazekas scales. As expected, WMHs with higher Fazekas scale has lower FA, higher MD, AD and RD. These changes of DTI metrics may be caused by gliosis, demyelination, fluid accumulation and axonal loss among others [22]. However, some differences existed in the correlation coefficients of different regions, which means that WMH in different regions has certain heterogeneity.

Some studies have suggested that pathological differences exist between periventricular WMHs and deep WMHs [23–26]. Our results demonstrated that microstructural changes around the frontal periventricular WMHs were significantly different from those in other regions. There were significantly lower FA values, but higher AD, RD, and MD values in frontal periventricular WMHs compared to other regions. As there were no such differences as indicated by the effect sizes in DTI metrics among different brain regions that featured normal white matter, therefore, these heterogeneities were likely not due to anatomical heterogeneities. These results underscore the pathological heterogeneity of WMHs in different regions. Previous studies with magnetization transfer imaging also showed a lower magnetization transfer ratio in frontal periventricular WMHs compared to parieto-occipital periventricular WMHs [19, 20]. Spilt et al. [19] suggested that age-related periventricular WMH around the frontal and occipital horns have different etiologies.

Some pathological studies suggested that periventricular WMHs occurred because of fluid accumulation within the extracellular space that was related to the loss of ventricular ependyma [27–29]. Although increases of interstitial water content might have led to significant increase in MD, these changes could not explain the more immediate changes in RD values, compared to AD values. Our results showed that the decrease of FA and the increase of RD and MD in the periventricular WMHs were more significant than those in the other parts of WMHs. In the demyelinating animal model, the RD of the lesion was significantly higher than that of the normal white matter, while the AD was unchanged [30]. These uncoordinated changes in AD and RD were also observed in patient with multiple sclerosis [31]. Immunohistochemical findings also revealed that periventricular WMHs demonstrated more severe demyelination than deep WMHs [27]. A past neuropathological study found a stronger relationship between the extent of WMHs on MRI and the extent of myelin rarefaction in the frontal lobe, compared to the parietal lobe [32]. Another evidence is the lower T1-weighted signal of WMH around frontal horns [19], which is consistent with the “black hole” of demyelinating pathological changes [33]. Therefore, we believe that demyelination contributed to the pathological changes of frontal periventricular WMH. DTI, as a non-invasive technology, has the potential to classify WMH based on pathological changes, which will provide great value for future research.

Some studies [9–11] have elucidated links between cognitive decline and periventricular WMHs in the frontal lobe, parieto-occipital lobe, and other parts of the brain. However, the underlying pathological mechanisms of WMHs in different regions that might explain cognitive decline (other than anatomical locations) remain scarcely studied [9]. A recent DTI study [10] showed that, in patients with hypertension, anterior thalamic radiations, the superior longitudinal fasciculus, and the forceps minor had significantly lower FA and significantly higher MD, AD, and RD values; these changes corresponded with measures of cognitive impairment. In Duering’s study [13] of cerebral autosomal dominant arteriopathy with subcortical infarcts and leukoencephalopathy (CADASIL), anterior thalamic radiations and frontal forceps were also considered key sites of cognitive impairment. These results suggested that frontal WMHs may contribute more to functional impairments. Frontal white matter microstructural changes began before the appearance of hyperintensities that could be found using conventional MRI. They appeared at an earlier age and demonstrated less age-related progression than WMHs in other regions [12]. This may lead to inconsistencies between the extent of WMHs and their respective pathological severities. The underlying pathological changes may be severe, even with little volumetric expansion. These findings suggested that even Fazekas grade 1 WMHs around the frontal lateral ventricle may correspond to clinically significant pathological changes. We suggest that future studies should consider not only the volume of WMH, but also their pathological severities.

Our study still has some limitations. First, normal white matter FA values decreased with age, while MD values increased with age. Average, our control group was significantly younger than patient group. However, the differences between WMHs and standard DTI metrics were greater than the normal variation observed with age [34]. We try to eliminate this effect by using age and gender as covariates. Second, to minimize the influence of DTI parameters caused by anatomical variations, we selected ROIs within identical white matter tracts, according to the color maps; however, variations may still exist for heterogeneity. Third, since the structural changes reflected by DTI parameters were non-specific and may have been affected by many factors, relevant pathological changes remain poorly elucidated. Fourth, the Fazekas classification method of WMH is not a quantitative evaluation method and may be affected by the different observer. However, the quantitative evaluation method is also limited by the division of ventricular and deep WMH and not a widely accepted approach [8].

## Conclusions

Our results showed that pathological processes differed between frontal periventricular WMHs and WMHs of other regions. These differences may be due to more severe demyelination in the periventricular frontal white matter tracts. DTI has the potential to classify WMH based on pathological changes non-invasively. Future studies should consider pathological heterogeneity as a factor to better explore the relationships between WMHs and cognitive impairment.

## Supplementary Information

**Additional file 1.**

## Data Availability

All data generated or analysed during this study are included in this published article and its supplementary data files (Rawdata-rv2 .xlsx).

## Abbreviations

WMHs: White matter hyperintensities
T2WI: T2-weighted imaging
FLAIR: Fluid-attenuated inversion recovery
MRI: Magnetic resonance imaging
DTI: Diffusion tensor imaging
FA: Fractional anisotropy
MD: Mean diffusivity (MD)
AD: Axial diffusivity
RD: Radial diffusivity
ROIs: Regions of interest (ROIs)
pFL: Periventricular frontal lobe
pOL: Periventricular occipital lobe
pPL: Periventricular parietal lobe
dCO: Deep centrum ovale
CADASIL: Cerebral autosomal dominant arteriopathy with subcortical infarcts and leukoencephalopathy
